# Analysis of the gut-specific microbiome from field-captured tsetse flies, and its potential relevance to host trypanosome vector competence

**DOI:** 10.1186/s12866-018-1284-7

**Published:** 2018-11-23

**Authors:** Bridget C Griffith, Brian L Weiss, Emre Aksoy, Paul O Mireji, Joana E Auma, Florence N Wamwiri, Richard Echodu, Grace Murilla, Serap Aksoy

**Affiliations:** 1 Department of Epidemiology of Microbial Diseases, Yale School of Public Health, New Haven, CT USA; 2 Present Address: Division of Epidemiology and Community Health, School of Public Health, University of Minnesota, Minneapolis, MN USA; 3 Present Address: Department of Entomology, University of California Riverside, Riverside, CA USA; 4 Biotechnology Research Institute, Kenya Agricultural and Livestock Research Organization, Kikuyu, Kenya; 5 Department of Biology, Faculty of Science, Gulu University, Gulu, Uganda

**Keywords:** Tsetse fly, *Glossina*, Symbiont, *Wigglesworthia*, *Sodalis*, Microbiota, African trypanosome, Metagenomics

## Abstract

**Background:**

The tsetse fly (*Glossina* sp.) midgut is colonized by maternally transmitted and environmentally acquired bacteria. Additionally, the midgut serves as a niche in which pathogenic African trypanosomes reside within infected flies. Tsetse’s bacterial microbiota impacts many aspects of the fly’s physiology. However, little is known about the structure of tsetse’s midgut-associated bacterial communities as they relate to geographically distinct fly habitats in east Africa and their contributions to parasite infection outcomes. We utilized culture dependent and independent methods to characterize the taxonomic structure and density of bacterial communities that reside within the midgut of tsetse flies collected at geographically distinct locations in Kenya and Uganda.

**Results:**

Using culture dependent methods, we isolated 34 strains of bacteria from four different tsetse species (*G. pallidipes*, *G. brevipalpis*, *G. fuscipes and G. fuscipleuris*) captured at three distinct locations in Kenya. To increase the depth of this study, we deep sequenced midguts from individual uninfected and trypanosome infected *G. pallidipes* captured at two distinct locations in Kenya and one in Uganda. We found that tsetse’s obligate endosymbiont, *Wigglesworthia*, was the most abundant bacterium present in the midgut of *G. pallidipes*, and the density of this bacterium remained largely consistent regardless of whether or not its tsetse host was infected with trypanosomes. These fly populations also housed the commensal symbiont *Sodalis*, which was found at significantly higher densities in trypanosome infected compared to uninfected flies. Finally, midguts of field-captured *G. pallidipes* were colonized with distinct, low density communities of environmentally acquired microbes that differed in taxonomic structure depending on parasite infection status and the geographic location from which the flies were collected.

**Conclusions:**

The results of this study will enhance our understanding of the tripartite relationship between tsetse, its microbiota and trypanosome vector competence. This information may be useful for developing novel disease control strategies or enhancing the efficacy of those already in use.

**Electronic supplementary material:**

The online version of this article (10.1186/s12866-018-1284-7) contains supplementary material, which is available to authorized users.

## Background

Tsetse flies (*Glossina* spp.) are medically and agriculturally important insect vectors that transmit African trypanosomes, the causative agents of human and animal African trypanosomiases (HAT and AAT, respectively). Approximately 70 million people, living within an area of 1.55 million km^2^ in sub-Saharan Africa, are at risk for contracting HAT, which is almost always fatal if left untreated [1]. Additionally, AAT is estimated to cost African agriculture US$4.5 billion per year [2]. Disease preventing vaccines are currently unavailable due to the fact that trypanosomes continuously switch their antigenically distinct Variant Surface Glycoprotein coat to evade the mammalian host immune system [3]. Furthermore, drug resistance in parasites is increasing and can compromise the efficacy of treating infected patients chemotherapeutically [4]. Disease can be effectively controlled by reducing tsetse population densities during disease outbreaks. However, these practices are costly and labor-intensive and often rely on insecticide-treated devices or aerial sprays, which are environmentally unfriendly. Thus, novel disease control strategies, including those designed to reduce tsetse’s ability to transmit trypanosomes, can provide desirable cost-effective alternatives.

While only a small percentage of field-captured tsetse are infected with trypanosomes (reviewed in [5]), all individuals house enteric communities of indigenous, maternally transmitted symbiotic bacteria as well as bacteria acquired from the environment [6, 7]. Obligate *Wigglesworthia* [8] and commensal *Sodalis* [9] are vertically transmitted via maternal milk gland secretions to developing intrauterine larvae during tsetse’s unique viviparous mode of reproduction [10]. *Wigglesworthia* is found in all lab colonized and field-captured tsetse flies, and its symbiosis with the fly is ancient [11]. Female flies experimentally reared in the absence of obligate *Wigglesworthia* (via dietary supplementation of the blood meal with antibiotics) cannot support larval development and thus become reproductively sterile [12, 13]. *Wigglesworthia’s* genome encodes biochemical pathways responsible for the production of vitamins and co-factors that the fly cannot produce de novo and that are absent from its vertebrate blood-specific diet [14]. Correspondingly, metabolomic analyses [15] and dietary supplementation studies [16] indicate that loss of fecundity in symbiont-cured females results from decreased levels of essential nutrients (in particular B-Vitamins) that are necessary for tsetse to produce amino and nucleic acids. Obligate *Wigglesworthia* also mediates the development and function of its host’s immune system. Specifically, larvae that undergo development in the absence of this bacterium present a compromised cellular immune system as adults [17–19] and are unusually susceptible to infection with trypanosomes [20].

In contrast to *Wigglesworthia*, tsetse’s symbiosis with commensal *Sodalis* is relatively recent, and this bacterium can be cultured outside of tsetse and genetically modified [21]. These characteristics make *Sodalis* amenable as a potential candidate for paratransgenic control of trypanosome transmission [22]. While *Sodalis’* functional contribution to tsetse’s physiology is currently unknown, specific elimination of the bacterium from the fly results in a reduction in fly longevity [23]. Furthermore, *Sodalis* mediates tsetse’s susceptibility to infection with trypanosomes [24], and some field-based studies have demonstrated a positive correlation between the presence of *Sodalis*, and/or high *Sodalis* density, and parasite infection prevalence [7, 25–27]. Understanding factors that contribute to trypanosome infections in susceptible flies can lead to new vector control methods that are based on increasing parasite resistance phenotypes in natural population. Along with host genetic factors (such as hostile immune responses) that influence transmission dynamics, microbiota also influence pathogen transmission in vector insects, including tsetse [28].

In addition to maternally transmitted symbionts, wild tsetse flies also harbor a variety of environmentally-acquired bacteria in their guts. The taxonomic composition and density of these bacterial populations varies significantly within and between sympatric populations of tsetse composed of the same or different species. However, members of the phyla Bacteroidetes, Actinobacteria, Firmicuties and Beta- and Gamma-proteobacteria are found consistently within the gut of different tsetse species captured in geographically distinct locales [7, 29–31]. In most cases these transient microbes comprise less than 1% of tsetse’s cumulative enteric microbiota [7]. The origin of environmentally-acquired gut microbes, and their function as they relate to the physiology of their tsetse host, remains to be elucidated. In this study we employed culture dependent and independent methods in an effort to better define the core population of environmentally acquired bacteria that reside in the midgut of field-captured tsetse. Additionally, we investigated whether the taxonomic composition and relative abundance of distinct bacterial taxa correlates with environmental conditions and trypanosome infection status.

## Results

### Glossina collections

One-hundred and ninety-three tsetse flies, representing four species (*G. pallidipes*, *n* = 165; *G. fuscipes*, *n* = 4; *G. fusciplures*, *n* = 12; *G. brevipalpis*, *n* = 12) collected from five sites (Table 1), were used for this study. Of the 193 flies, 20 individuals representing four species (*G. pallidipes*, *n* = 11; *G. fuscipes*, *n* = 3, *G. fusciplures*, *n* = 3; *G. brevipalpis*, *n* = 3) were used to culture midgut bacteria in vitro (Table 1). All other experiments were performed using only *G. pallidipes*, of which 112 were used for culture independent characterization of midgut microbiota (Table 1), and 41 (21 trypanosome infected and 20 uninfected, as determined by microscopic analysis) were used to assess the correlation between symbiont density and trypanosome infection status.

**Table 1:** Geographic origin and sample size of tsetse species collected for this study

Geographic origin	*Glossina* spp.			
	*G. pallidipes*	*G. brevipalpis* ^*d*^	*G. fuscipleuris* ^*d*^	*G. fuscipes* ^*d*^
	sample size (% infected with trypanosomes)			
Shimba Hills, Kenya	58 (32.8%)^a^	12 (25%)	0	0
Trans Mara, Kenya	0	0	12 (25%)	0
Lake Victoria, Western Kenya	0	0	0	4 (0%)
Nguruman Escarpment, Kenya	62 (14.5%)^b^	0	0	0
Murchison Falls, Uganda	45 (2%)^c^	0	0	0

^a^Culture dependent identification of gut bacteria, *n* = 11; culture independent identification of gut bacteria, *n* = 15; RT-qPCR based quantification of symbiotic bacteria, *n* = 32

^b^Culture independent identification of gut bacteria, *n* = 52; RT-qPCR based quantification of symbiotic bacteria, *n* = 10

^c^Culture independent identification of gut bacteria, *n* = 45

^d^All *G. brevipalpis*, *G. fuscipleuris* and *G. fuscipes* flies were used exclusively for culture dependent identification of gut bacteria

### Bacterial taxa cultured from the fly gut

Twenty-four fly midguts were subjected to identical bacterial isolation processes, and 20 (11 *G. pallidipes*, 3 *G. brevipalpis*, 3 *G. fuscipleuris* and 3 *G. fuscipes*) yielded culturable bacterial clones. Between one and four bacterial operational taxonomic units (OTUs) were identified from each fly, and 34 bacterial OTUs were isolated in total (Table 2, Additional file 1: Table S1). The 16S *rRNA* gene from all 34 isolates was amplified and sequenced. Following the sequence alignment, members of 14 different genera were identified (Table 2). The most common isolate was *Bacillus* sp*.,* which was identified from 10 (50.0%) individuals. Both *Bacillus* and *Staphylococcus* were isolated from individuals in all three collection sites. *G. fuscipleuris* collected in Trans Mara, Kenya had the greatest number of genera represented (6), while only one genus was isolated from Shimba Hills *G. brevipalpis*. Of the 20 flies examined, 10 housed one culturable OTU, while 10 housed two or more (Table 2).

**Table 2:** Bacteria isolated from tsetse fly midguts using culture dependent techniques

Geographic origin	*Glossina* spp.	fly#	bacterial taxa identified
Shimba Hills, Kenya	*G. pallidipes*	1	*Staphylococcus* spp.
		2	*Xylella* spp., *Staphylococcus* spp.
		3	*Staphylococcus* spp.
		4	*Agrococcus* spp., *Arthrobacter* spp.
		5	*Bacillus* spp.
		6	*Staphylococcus* spp., *Bacillus* spp.
		7	*Staphylococcus* spp.
		8	*Staphylococcus* spp.
		9	*Enterobacter* spp., *Bacillus* spp.
		10	*Exiguobacterium* spp., *Bacilus* spp.
		11	*Exiguobacterium* spp., *Bacilus* spp.
	*G. brevipalpis*	1	*Bacillus* spp.
		2	*Bacillus* spp.
		3	*Bacillus* spp.
Trans Mara, Kenya	*G. fuscipleuris*	1	*Staphylococcus* spp., *Bacillus* spp.
		2	*Kocuria* spp., *Microbacterium* spp.
		3	*Exiguobacterium* spp., *Sinomonas* spp.
Lake Victoria, Western Kenya	*G. fuscipes*	1	*Bacillus* spp., *Oceanimonas* spp., *Microbacterium* spp., *Staphylococcus* spp.
		2	*Arthrobacter* spp., *Aeromonas* spp., *Providencia* spp., *Bacillus* spp.
		3	*Pantoea* spp.

Out of the 34 isolates, 21 belonged to the phylum Firmicutes, 6 to Proteobacteria and 7 to Actinobacter. With the exception of *G. brevipalpis*, in which only one bacterial genus was found, representative isolates of the above three phyla were found in each tsetse species (Additional file 1: Table S1).

### Tsetse gut-associated microbes identified via culture independent methods

To better define the population of bacteria that reside transiently in tsetse’s midgut, we used PCR to generate barcoded 16S rDNA libraries from 112 *G. pallidipes* midguts. These libraries were subsequently pooled and deep sequenced on the Illumina MiSeq platform. A total of 21,728,153 reads were obtained from the 112 samples. After quality filtering, a total of 16,635,470 sequences were obtained (average 148,531 sequences per sample) and entered into the QIIME software pipeline for alignment and taxonomic assignment.

### Midgut microbial diversity across multiple G. pallidipes populations

**Fig. 1:**
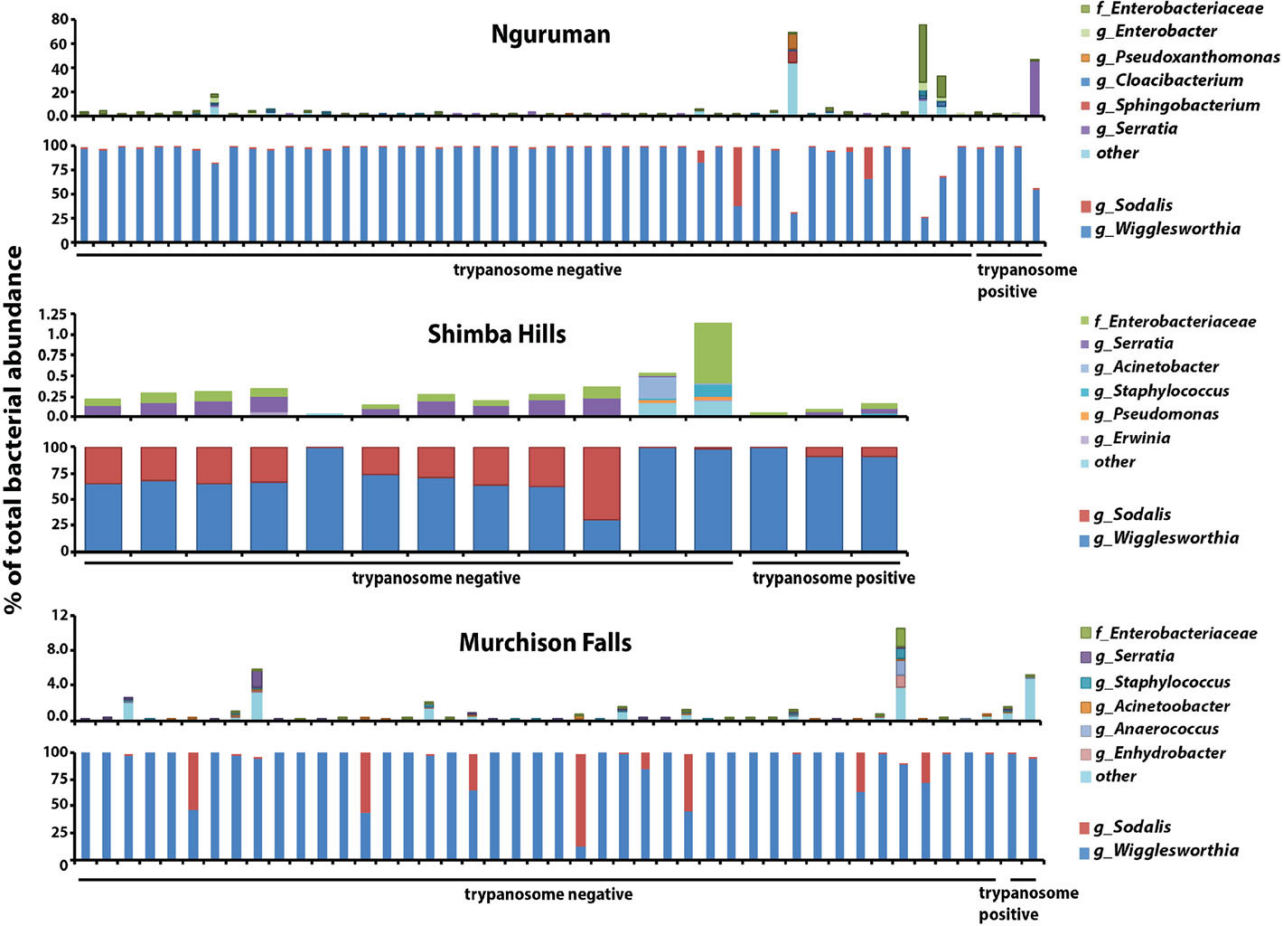
Taxonomic composition and % abundance of bacteria found in midguts of uninfected and trypanosome infected *G. pallidipes* caught in Nguruman, Kenya, Shimba Hills, Kenya and Murchison Falls, Uganda. Data collected from each location is exhibited on two graphs. The top and bottom graphs show the % abundance of total midgut bacteria that are composed of environmentally acquired and maternally transmitted OTUs, respectively. Individual flies assayed from each site are represented by a distinct bar on each graph

Using 16 s rDNA deep sequencing, we identified bacterial OTUs within individual *G. pallidipes* trapped in Nguruman, Shimba Hills and Murchison Falls. Figure 1 presents three sets of graphs, each of which shows data from one of the different trapping sites. Individual bacterial OTUs are indicated as % abundance of the total population present in each individual fly gut. *Wigglesworthia* and *Sodalis* are presented on the bottom graph, while the top graph presents the next six most abundant environmentally acquired bacteria (averaged across samples and excluding *Wigglesworthia* and *Sodalis*). Results are presented in this manner in an effort to better visualize the community of exogenous, environmentally acquired bacteria detected in the gut of *G. pallidipes* collected from ecologically distinct habitats. In a previous study we found that *Wigglesworthia* represented > 99% of the total bacteria found in the midgut of *G. fuscipes* captured in Murchison Falls [7]. In an effort to more completely resolve the population of environmental bacteria found in the midgut of *G. pallidipes* used in this study, we treated the 16 s rDNA amplicons with *Eco*RV to remove *Wigglesworthia*-specific sequences. As such, % abundance data presented here excludes *Wigglesworthia* sequences removed by this treatment.

At Nguruman Escarpment in south-central Kenya, the 6 most abundant environmentally acquired taxa were comprised of members of the family Enterobacteriaceae (including the genera *Enterobacter* and *Serratia*), as well as members from the genera *Pseudoxanthomonas, Cloacibacterium,*and *Sphingobacterium*. In *G. pallidipes* captured on the Kenya’s east coast in Shimba Hills National park, the bacterial population was represented by members of the family Enterobacteriaceae (including the genus *Serratia*) and members from the genera *Acinetobacter*, *Staphylococcus*, *Pseudomonas* and *Cloacibacterium.* Finally, the 6 most abundant environmentally acquired taxa found in guts of *G. pallidipes* collected in Murchison Falls, Uganda included members of the family Enterobacteriaceae (including the genus *Serratia*) as well as members from the genera *Staphylococcus*, *Acinetobacter*, *Anaerococcus* and *Enhydrobacter* (Fig. 1).

In addition to characterizing the population of environmentally acquired bacteria that reside in field-captured *G. pallidipes*, we also used our deep-sequencing reads as a source of data to quantify the density of maternally transmitted symbionts found in these flies. At all three trapping locations, obligate *Wigglesworthia* represented the majority of bacterial OTUs by % abundance in all individuals (Nguruman, 91.99%; Shimba Hills, 76.67%; Murchison Falls, 90.26%; Fig. 1). *Sodalis*, a commensal tsetse symbiont, comprised an average of 2.37%, 23.05% and 8.09% of the total bacteria present in *G. pallidipes* sampled in Nguruman, Shimba Hills and Murchison Falls, respectively (Fig. 1). The averages for each sampling site, also presented as % of total bacterial abundance, are displayed in Additional file 1: Figure S1.

### Diversity measures

**Fig. 2:**
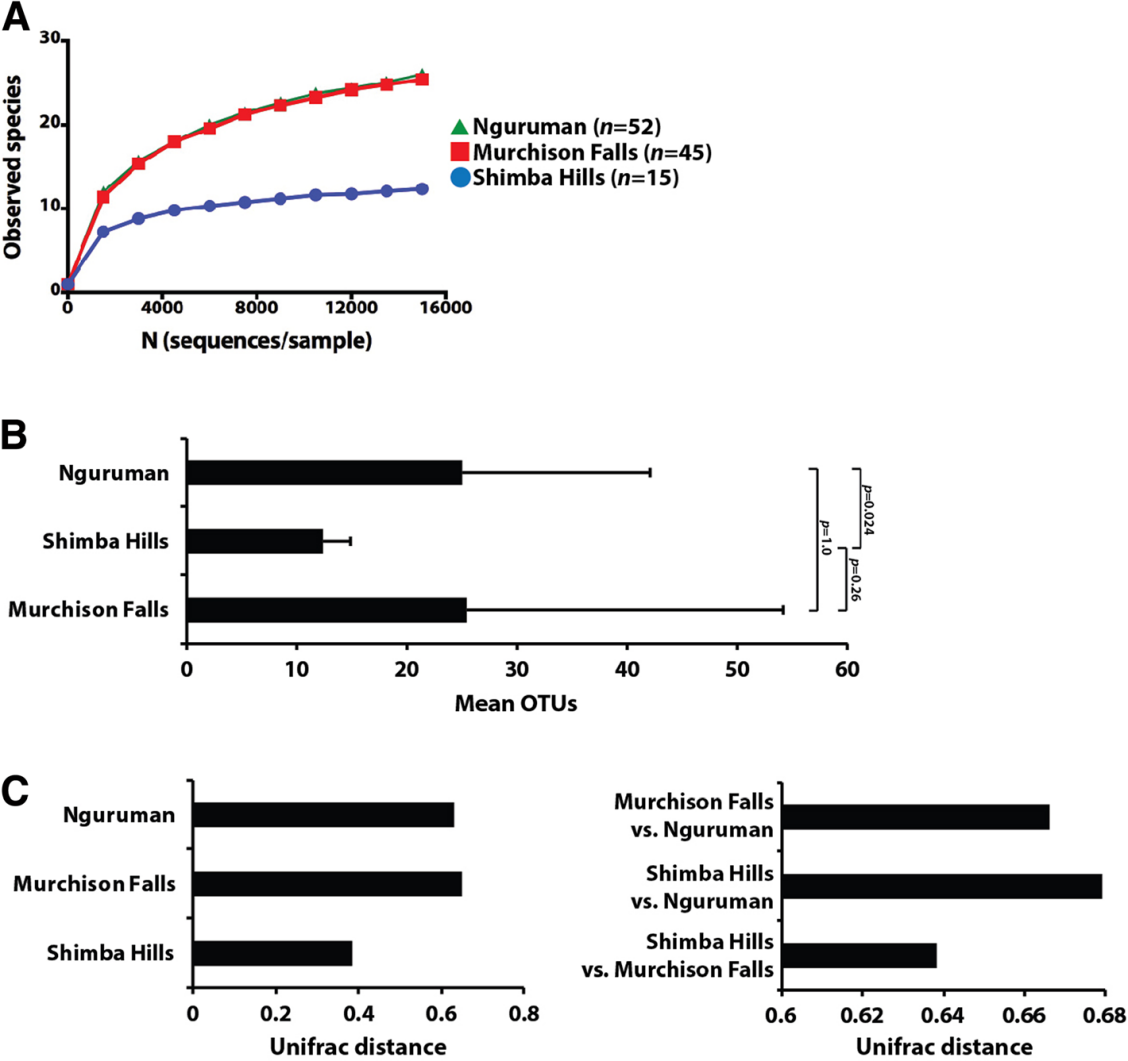
Measurement of bacterial α and β-diversity in midguts of *G. pallidipes* captured in Shimba Hills, Nguruman and Murchison Falls. Plots (**a**) and (**b**) present bacterial α-diversity (indicative of species richness), which was measured using the ‘observed species metric’. **a** Rarefaction curves demonstrate the analysis achieved adequate sequencing depth and OTU discovery. **b** At a 95% confidence level (*α* = 0.05), a statistically significant difference in species richness was observed between flies collected from Shimba Hills vs. Murchison Falls (*p* = 0.024). Plot (**b**) shows bacterial β-diversity measured using the unweighted UniFrac metric and Bray Curtis method. **c** Average UniFrac distance within each collection site (left graph) and between each collection site (right graph). β-diversity was statistically significantly different between *G. pallidipes* captured in Shimba Hills and Murchison Falls (nonparametric paired t-test; *p* = 0.028). β-diversity between the other two sites was not significantly different at a 95% confidence level

We used the “observed species” metric to measure α-diversity (species richness) of bacteria found in guts of *G. pallidipes* collected from all three sites. The rarefaction curve leveled off at 6000 sequences per sample, indicating that an adequate sequencing depth and OTU discovery was achieved (Fig. 2a). At a 95% confidence level (*α* = 0.05), we observed statistically significant differences in α-diversity between one of the three study sites (Fig. 2b). Specifically, Shimba Hills had the lowest species richness (mean OTUs, 12.38 ± 2.45), which was statistically significant different from Nguruman (mean OTUs, 25.05 ± 17.11; *p* = 0.024). Shimba Hills was not significantly different from Murchison Falls (mean OTUs, 25.5 ± 28.73; *p* = 0.261), and Murchison Falls and Nguruman were not significantly different from each other (*p* = 1; Fig. 2b).

We next used the unweighted UniFrac metric, and the Bray Curtis method, to measure β-diversity of bacteria within and between *G. pallidipes* individuals collected at the three geographic regions. Figure 2c displays the average UniFrac distance within each collection site (left graph) and between each collection site (right graph). Using a nonparametric paired t-test, significant differences in the β-diversity among the *G. pallidipes* flies trapped in Shimba Hills and Murchison Falls (*p* = 0.028) were observed. The β-diversity of the other two sites was not significantly different at a 95% confidence level.

### Tsetse’s microbiota and trypanosome infection status

The gut microbiota of arthropod disease vectors influences their host’s capacity to transmit pathogenic microorganisms [28, 32, 33]. In tsetse, the obligate mutualist *Wigglesworthia* plays important roles in defining the ability of trypanosomes to establish an infection in tsetse’s gut. More specifically, this bacterium mediates the production of trypanocidal Peptidoglycan Recognition Protein LB (PGRP-LB) [34] as well as the development and function of a midgut barrier called the peritrophic matrix [20]. A correlative relationship also exists between the presence of *Sodalis* and increased susceptibility of tsetse to infection with trypanosomes (reviewed in [6]), although the mechanistic basis of this interaction remains unknown.

**Fig. 3:**
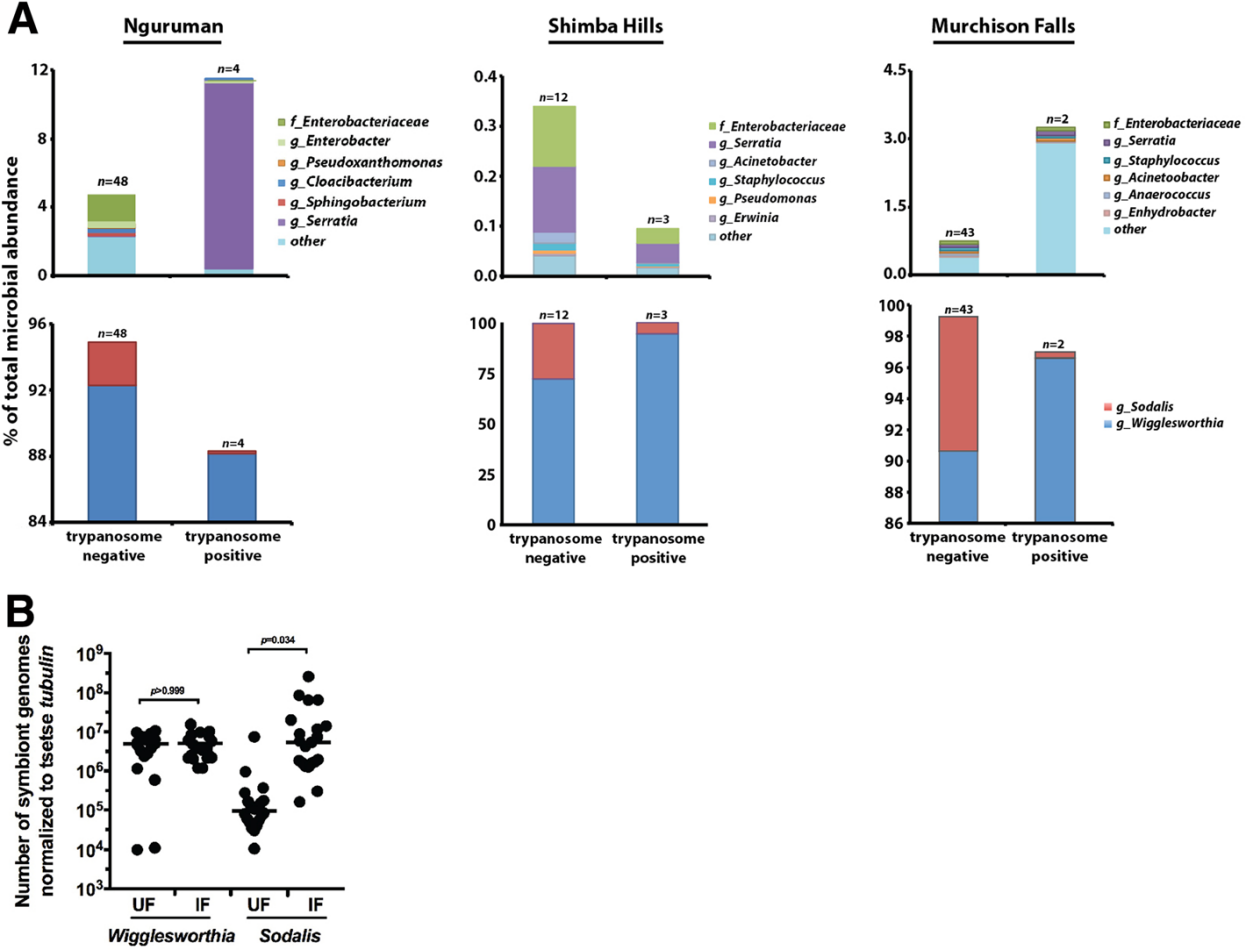
Average taxonomic composition and % abundance of environmentally acquired and symbiotic bacteria found in midguts of uninfected and trypanosome infected *G. pallidipes* captured in Nguruman, Kenya, Shimba Hills, Kenya and Murchison Falls, Uganda. **a** Community structure of environmentally acquired bacteria (top set of graphs), and maternally transmitted symbionts (bottom set of graphs), identified from midguts of field-captured *G. pallidipes*. Data on the top and bottom graphs are presented as % abundance of the total (100%) bacterial population present in trypanosome uninfected and infected flies from each geographic location. The range of values on the Y-axes on the bottom set of graphs (maternally transmitted symbionts) is different so that low density *Sodalis* populations are visible. **b** Real-time quantitative PCR-based determination of *Wigglesworthia* and *Sodalis* densities in uninfected (UF) and trypanosome infected (IF) *G. pallidipes* trapped in Nguruman and Shimba Hills. Sample data derived from uninfected and parasite infected flies captured at each location was pooled so as to present an overall picture of the relationship between symbiont density and trypanosome infection status. Statistical significance of the data was determined by one-way ANOVA using GraphPad Prism software, v. 7

Herein we compared the taxonomic structure of bacterial communities that reside in guts of trypanosome uninfected versus infected *G. pallidipes* captured in Shimba Hills, Nguruman and Murchison Falls. To do so we separated our cumulative 16S data from all 3 locations (displayed in Fig. 1 and Additional file 1: Figure S1) into uninfected and trypanosome infected pools, and then averaged the density of the six most abundant environmentally acquired microbes, as well as *Wigglesworthia* and *Sodalis*, found in each of the pools. With respect to environmentally acquired enteric bacteria, *Serratia* spp. were dominant in parasite infected *G. pallidipes* collected in Nguruman, and in both uninfected and infected individuals from Shimba Hills. Additionally, other members of the family *Enterobacteriaceae* were also found in relatively high numbers in uninfected flies from both of these locations, and in parasite infected flies from Shimba Hills (Fig. 3a, top graphs). Unlike their counterparts found in Nguruman and Shimba Hills, *G. pallidipes* captured in Murchison Falls housed mainly ‘other’ bacteria (could not be resolved to family level) in their guts, regardless of trypanosome infection status (Fig. 3a, top graphs).

‘Similarity’ tests, performed to take into account both weighted (takes into account species abundance) and unweighted (takes into account bacterial taxonomic structure) distance matrices, indicated that the taxonomic composition of environmentally acquired enteric microbes was significantly different between trypanosome infected and uninfected *G. pallidipes* collected in Shimba Hills (unweighted *p*-value = 0.01, *R* = 0.55; weighted *p*-value = 0.02, *R* = 1). Conversely, the taxonomic composition of environmentally acquired enteric microbes was not significantly different between trypanosome infected and uninfected *G. pallidipes* collected in Nguruman (unweighted *p*-value = 0.14, *R* = 0.14; weighted *p*-value = 071, *R* = − 0.16) nor Murchison Falls (unweighted *p*-value = 0.64, *R* = − 0.09; weighted *p*-value = 0.08, *R* = 0.41). Taken together, these findings suggest that the taxonomic structure and density of environmentally acquired enteric bacteria differs based on whether or not the host fly harbors an infection with trypanosomes. A more robust analysis, making use of a larger population of infected flies, is required in order to definitively correlate the taxonomic structure of environmentally acquired enteric bacteria with trypanosome infections outcomes in field-captured tsetse.

We next used the 16S rDNA data to investigate the relationship between symbiont density and trypanosome infection status. As expected we found that *Wigglesworthia* comprised the majority of bacterial taxa present in guts of uninfected and infected *G. pallidipes* from all three sites (Fig. 3a, bottom graphs). Interestingly, we observed that *Sodalis* was more abundant in guts of uninfected flies compared to trypanosome infected individuals captured in all three locations (Fig. 3a, bottom graphs). These findings contradict those from previous studies, which correlate high *Sodalis* density with increased trypanosome infection prevalence [25–27]. We thus investigated this finding in more depth by using real-time quantitative PCR to determine the density of *Wigglesworthia* and *Sodalis* in a larger sample size of trypanosome uninfected and infected *G. pallidipes*. We did not have any infected flies remaining from Murchison Falls, hence this experiment was performed with individuals collected in Nguruman and Shimba Hills. When the data from the two sites was combined, we observed no significant difference in the *Wigglesworthia* density between uninfected and infected individuals. However, *Sodalis* was significantly more abundant in trypanosome infected flies compared to their uninfected counterparts (Fig. 3b).

## Discussion

In the present study we used culture dependent and independent methods to characterize the enteric microbiota found in tsetse flies, collected from three geographically and environmentally distinct locations, in an effort to better understand their contributions to tsetse’s vector competence. Culture dependent analysis demonstrated that specific bacterial taxa resident within tsetse’s midgut can be grown in vitro using previously described methods [31]. Further, more rigorous analysis using next-generation sequencing technology revealed that bacterial taxa present in the midgut of one tsetse species, *G. pallidipes*, varied depending on the site at which flies were collected. This habitat-based variation in microbiota composition was reflected in the abundance of both maternally transmitted obligate and commensal symbionts as well as environmentally acquired bacteria. Additionally, clear differences were present in the density and taxonomic composition of bacteria found in guts of trypanosome infected compared to uninfected flies of the same *Glossina* species collected from the same location. Overall, these results suggest that the environment in which tsetse reside plays an important role in the composition of the fly’s midgut microbiome. Furthermore, the composition of tsetse’s midgut microbiota may relate to the fly’s ability to transmit pathogenic African trypanosomes. Additional research is required to determine the role of maternally transmitted and environmentally acquired bacteria as they relate to the physiology of their tsetse host.

In this study, 34 bacterial isolates were successfully cultured, of which 21 belonged to the phylum Firmicutes, six to Proteobacteria and seven to Actinobacter. With the exception of *G. brevipalpis*, from which only one bacterial genus was cultured, representative isolates of the above three phyla were found in each tsetse species. Both *Bacillus* and *Staphylococcus* were isolated from individuals in all three collection sites. In this study, Firmicutes and *Bacillus* were the dominant phylum and genus, respectively. Three previous studies [29–31] have employed culture dependent methods to investigate the microbial composition of tsetse midguts from fly populations geographically distinct from the ones used here in. Bacterial strains from the Firmicutes, Proteobacteria and Actinobacter were identified in each case, although the dominant phyla observed in each study was variable. This variability could be due to the fact that different culture conditions were utilized in each case. In this study three different types of media were tested, and all samples were grown at room temperature in anaerobic and ambient oxygen concentrations. Thus, it appears as though the diversity and relative abundance of microbes that can be cultured from tsetse’s gut depends upon several factors, including the environment in which the fly lives as well as the conditions in which the isolates were grown. Further studies that engage different culture conditions should be performed in an effort to identify additional culturable bacteria from the tsetse fly gut. These bacteria may be of particular importance because they can potentially be genetically modified and/or trans-located between tsetse species or between field-captured flies and insectary-reared individuals that harbor a different microbiota. Such taxa may be useful for performing functional experiments that will provide insight into how tsetse’s microbiota modifies their host’s physiology, including trypanosome vector competence. Additionally, culturable isolates may serve as candidates in a control strategy in which tsetse are colonized with recombinant bacteria that express anti-trypanosomal effector molecules. These ‘paratransgenic’ flies could exhibit a reduced ability to successfully transmit parasites between mammalian hosts [22, 35, 36].

Previous experiments performed to characterize tsetse’s midgut microbiome have lacked the illustrative power of comparing culture dependent and independent analyses in the same study population. This experimental shortcoming is important for two reasons. First, it fails to address what proportion of the total bacterial population is represented by culturable organisms. Secondly, it does not allow for the identification of taxa that cannot be cultured under the specific experimental conditions used in the study. As such, the vast majority of bacteria present in the niche are left unidentified. Recently, deep sequencing technology, based on the Illumina MiSeq platform, was used in an attempt to acquire a more in-depth overview of the microbiota found in guts of tsetse collected in Uganda [7]. This study successfully revealed that tsetse does house a more taxonomically complex gut microbiota than that identified via culture dependent methods and 16 s rRNA clone libraries. However, determining a comprehensive picture of the population structure of environmentally acquired microbes present in samples from the Ugandan study was likely obfuscated by the fact that obligate *Wigglesworthia* represented greater than 99% of the cumulative OTUs observed. This impediment was partially circumvented in this study by digesting *Wigglesworthia*-specific V4 PCR products with *Eco*RV endonuclease prior to library sequencing. This treatment succeeded in eliminating a significant proportion of *Wigglesworthia* reads (14.0% of total OTUs), thus allowing for a more comprehensive view of other microbial taxa resident in tsetse midguts analyzed in this study.

Results from this study indicate that adult tsetse flies house a taxonomically complex population of bacteria in their gut. The biological mechanisms that underlie colonization of tsetse’s midgut by environmental bacteria requires further investigation. However, the dynamics of this process are presumably different from that which occurs in other well-studied insect models. For example, free-living larval fruit flies and mosquitoes acquire nutrients directly from in their resident environment, which is rotting organic matter and fetid water, respectively. As such, immature stages of these insects house a robust gut microbiota [37–39]. Conversely, immature tsetse undergo their entire larval development within their mother’s uterus. This environment is devoid of environmental microbes, and during this time larvae are exposed exclusively to low densities of maternally-transmitted *Wigglesworthia, Sodalis* and in some cases *Wolbachia* [40]. Tsetse flies acquire food from their environment only during the adult stage of their lifecycle. Following eclosion teneral adults seek a vertebrate host that can serve as a source of blood, which is sterile unless the animal is septic. Prior to imbibing a meal, tsetse probe their host’s skin in an effort to locate an effectual bite site, and during this process the fly’s mouthparts likely come into direct contact with a diverse assemblage of bacteria that reside in this environment. Different tsetse species exhibit distinct host preferences [41], and this results in exposure to vertebrate host-specific microbiotas. Presumably all or a proportion of these bacteria represent the population that colonizes tsetse’s gut. Additionally, adult tsetse feed multiple times, so the diversity of environmentally acquired bacteria that resides in a fly’s gut likely increases as a function of age (which is a parameter that we did not control for in this study). These and other ecological variables (e.g., temperature, humidity, temporal availability of blood, etc.) cumulatively account for the variability in the taxonomic structure and density of bacterial communities we observed in *G. pallidipes* captured at geographically isolated locations. Further experimental studies are required to decipher the dynamic mechanisms that underlie colonization of tsetse’s gut by bacteria that reside in the fly’s environment.

To date, no experimental evidence exists to suggest that environmentally acquired bacteria mediate trypanosome infection outcomes in tsetse. However, studies performed using other insect vector model systems indicate bacteria of this nature do modulate host vector competence. For example, *Anopheles gambiae*, which is the principle vector of human malaria (*Plasmodium* sp.), harbors a taxonomically diverse assemblage of gut-associated bacteria [42]. Among this population, a commensal from the genus *Enterobacter* (designated ‘*Enterobacter* sp. Z, or *Esp*_Z) exhibits direct anti-*Plasmodium* activity via the production of toxic reactive oxygen intermediates [43]. *Plasmodium*-susceptible laboratory lines of *A. gambiae* were rendered highly resistant to parasite infection when they had been inoculated with *Esp*-Z prior to exposure to an infectious blood meal [43]. Additionally, *A. gambiae* can harbor *Serratia marcescens* in its gut, and this bacterium is also associated (through a currently unknown mechanism) with a *Plasmodium*-refractory host phenotype [44, 45]. Both *Enterobacter* and *Serratia* strains were identified within the gut of field-captured tsetse (this study, and [31]). Additionally, *Serratia* was found to reside at low densities within the gut of colonized flies. The functional relationship between these bacteria (as well as other environmentally-acquired commensals) and tsetse’s competence as a vector of African trypanosomes remains to be elucidated.

Tsetse’s well-studied maternally-transmitted symbionts mediate host vector competence through indirect mechanisms. This study, along with previous ones [26, 46], suggest that tsetse populations that harbor *Sodalis* are more likely to be infected with trypanosomes than are flies that do not harbor this bacterium, or harbor the bacterium at a relatively low density [7]. Although the mechanism that underlies this phenomenon is currently not well understood, it may involve the inhibition of tsetse-derived trypanocidal lectins by N-acetyl glucosamine, the latter of which is produced when chitin is degraded by *Sodalis* secreted chitinase [24]. A more recent theory suggests that a *Sodalis*-hosted prophage induces the production of potent antimicrobial effector molecules in trypanosome challenged flies [47]. Tsetse’s obligate endosymbiont, *Wigglesworthia*, also indirectly modulates trypanosome infection outcomes in tsetse. More specifically, peptidoglycan (PGN), which is shed by *Wigglesworthia* as the bacteria multiplies, induces bacteriocytes to produce PGRP-LB. PGRP-LB presents catalase activity that degrades free PGN, which would otherwise induce tsetse immune pathways that damage *Wigglesworthia*. Interestingly, PGRP-LB also exhibits anti-trypanosomal activity and acts as a first line in defense against parasite infections in the gut [34]. The quantity of this protein is proportional to *Wigglesworthia* density [48], and flies that harbor more of this bacterium likely exhibit greater resistance to parasites. In addition, flies that undergo intrauterine larval development in the absence of *Wigglesworthia* exhibit higher susceptibility to trypanosomes as adults [12, 20]. An important difference between wild-type and *Wigglesworthia*-free adults relates to the integrity of their peritrophic matrix (PM). *Wigglesworthia*-free adults fail to produce a structurally robust PM, which is a sleeve-like barrier that lines the fly midgut and separates immuno-reactive epithelial cells from the parasite-containing blood bolus. As such, the midgut of PM compromised flies respond immunologically to the presence of parasites earlier in the infection process than in wild-type individuals that house an intact PM. This irregular immune response exhibits limited trypanocidal activity, thus resulting in the parasite-susceptible phenotype exhibited by these flies [20]. In addition, *Wigglesworthia*-free flies present a compromised cellular immune system and lack a functional melanization cascade [19]. Whether this immune pathway influences trypanosome infection establishment in tsetse’s gut remains to be determined.

## Conclusions

In conclusion, findings from this study enhance our understanding of the relationship between the environment in which distinct tsetse populations reside, the structure of enteric bacterial communities and factors that mediate the establishment of trypanosome infections. Additionally, bacteria cultured during this study will contribute to our repertoire of culturable insect gut bacteria that may potentially find application in microbe-driven modulation of vector competence in tsetse and related flies. These findings can find application in the design of tsetse vector control strategies using paratransgenic microbes to halt the transmission of trypanosomes within the tsetse fly. Future studies will aim at further investigating the relationship between host vector competence and the presence of environmentally acquired microbes.

## Methods

### Fly collections

Flies were trapped from May 2013 to August 2013 in Shimba Hills National Reserve, Kenya (4°15′21.99″S, 39°23′46.13″E), the Trans Mara, Kenya (1°16′59.89″S, 34°55′49.27″E), Western Kenya (on the eastern shore of Lake Victoria; 0°36′54.11″S, 34°05′25.15″E), Nguruman escarpment, Kenya (1°45′53.33″S, 36°01′02.55″E) and Murchison Falls, Uganda (2°16′13.5″E, 31°41′09.15″E). Tsetse were captured using Epsilon F3 Ngu cloth traps and biconical cloth traps baited with acetone and phenol, which are established tsetse olfactory attractants [25, 26]. *Glossina* were identified to species based on morphological criteria.

Depending on their intended experimental use (see details below), captured tsetse flies were either dissected in the field (and trypanosome infections status scored by microscopy analysis) or stored in ethanol (either whole flies or dissected midguts) for transport back to the lab at Yale University.

### Culture dependent identification of bacteria from midguts of field-captured tsetse

Culture dependent isolation of bacterial samples from the tsetse midgut (*G. pallidipes, G. brevipalpis, G. fuscipes, G. fuscipleuris*) was conducted according to the method outlined by Lindh and Lehane [31]. Stringent procedures were employed so as to process all samples under sterile conditions. Specifically, flies were surface sterilized via submersion for 5 min in 10% bleach, 5 min in 70% ethanol, and 5 min in sterile water (solutions were replaced regularly). All tools and dissection surfaces were sterilized after each dissection using 100% ethanol. Fly midguts were microscopically extracted and homogenized in 20ul sterile phosphate buffer saline (PBS) using a sterilize motorized pellet pestle. Homogenized midguts were serially diluted up to a 10^− 10^ dilution in sterile PBS, and a volume of each dilution was plated.

Bacteria were cultured in ambient and microaerophillic (using GasPak EZ Campy Container System; BD Bioscience) atmospheres and on three types of solid (agar-based) media: Brain-Heart Infusion supplemented with 10% blood (BHIB), Mitsuhashi-Mahamarosh (MM) or Luria-Bertani (LB). Sterile technique was confirmed by plating an aliquot of the PBS used for fly homogenization onto all three types of solid media followed by incubation in ambient or microaerophilic atmospheres.

Plates were examined daily for the presence of bacterial colonies, and those exhibiting a unique morphology were inoculated into 900ul of their respective media and grown overnight in a shaking incubator at 30 °C. Liquid cultures were preserved in 10% glycerol, flash frozen and stored at − 80 °C.

### 16S rDNA-based phylogenetic analysis of culturable bacteria

DNA was extracted from bacterial cells using a Qiagen DNAeasy blood and tissue kit. DNA isolated from bacterial clones was PCR amplified using primers (Additional file 1: Table S2) that specifically target the bacterial 16S rRNA gene. Bacterial clones (*n* = 38) were taxonomically characterized using this procedure. PCR products were sequenced at the DNA analysis facility on Science Hill at Yale University. The 16S rRNA sequence data was compared to catalogued sequences using the Basic Local Alignment Search Tool (BLASTn) and CLC Genomics Workbench v.6 (Venlo, Netherlands).

### PCR amplification of the bacterial 16S rRNA gene and Illumina library preparation

Genomic DNA was extracted from the guts of field-captured *G. pallidipes* (*n* = 112) using either a DNEasy Blood and Tissue Kit (Qiagen) or a Masterpure-Complete DNA and RNA Purification Kit (EpiCentre). Negative control extractions were performed on reagents from the Qiagen and Epicentre DNA extraction kits, as well as the New England Biolabs Phusion PCR kit (see below). Samples were then PCR amplified using barcoded Illumina fusion primers (generously donated by Dr. Howard Ochman) that specifically target a 300 bp region of the V4 hypervariable region of the bacterial 16S rRNA gene [49]. Primers used to generate 16S rDNA products can be found at (ftp://ftp.metagenomics.anl.gov/data/misc/EMP/SupplementaryFile1_barcoded_primers_515F_806R.txt). Each sample was assigned a unique 12 base pair Golay barcode located on the 806R primer. Each PCR reaction was carried out in 30ul of volume containing 1ul of DNA, 0.2ul of Phusion Taq (New England Biolabs), 6ul of 5× reaction buffer, 0.6ul of 10 mM dNTPs, 0.75ul of 10uM forward and reverse primers and 20.7uls of dH_2_0. Cycling conditions were 1 min of initial denaturation at 98 °C followed by 35 cycles at 98 °C for 10 s, 54 °C for 15 s, 72 °C for 15 s and a final elongation step at 72 °C for 2 min. PCR reactions were performed in triplicate, pooled together and analyzed on a 2% agarose gel. The final PCR product, which was 384 bp in length, included 5′ and 3’ Illumina barcodes that flanked the paired 300 bp target region.

A previous characterization tsetse’s gut microbiota revealed that the population is dominated (> 99% of the total population) by obligate *Wigglesworthia* [7]. This phenomenon significantly reduced, or may have entirely excluded, detection and accurate quantification of other less well represented microbes. In an effort to reduce *Wigglesworthia* bias and thus obtain a more in-depth representation of low density bacteria, we performed an *Eco*RV restriction endonuclease (New England BioLabs) digest of the pool of 16 s rDNA PCR fragments. This enzyme cuts within the V4 hypervariable region of *Wigglesworthia’s* 16S *rRNA* gene, but not this gene’s orthologue from *Sodalis* nor the majority of the environmental microbes identified previously [7]. Remaining PCR fragments were cleaned using Agencourt AMpure XP beads (Beckman Coulter), as per the manufacturer’s protocol. *Eco*RV digested DNA passed through the bead column and was thus excluded from downstream procedures. PCR products were quantified using the Qubit dsDNA High Sensitivity Assay (Life Technologies), and positive reactions were pooled at an equal molar concentration. In total, 16S rDNA sequences were pooled from 163 fly midguts. The pooled sample was sequenced on an Illumina MiSeq sequencing system at the Yale Center of Genome Analysis.

### Next generation sequencing data analyses

Reads were quality checked using FastQC software (http://www.bioinformatics.babraham.ac.uk/projects/fastqc/). The 16 s rRNA sequence dataset was demultiplexed and forward and reverse reads were paired using SeqPrep. In the event of a mismatched read, quality scores associated with each base were used to determine the appropriate pairing. In order to improve sequencing accuracy of low diversity samples, a phiX DNA control was added. To remove the phiX reads from the data set, paired reads were mapped to the phiX genome using Bowtie2 [50]. A list of reads not matching the phiX genome was generated using SamTools [51], and the resulting reads were separated from the phiX genome using QIIME software filter_fasta.py script [52]. Sequences were entered into the QIIME pipeline using the split_libraries_fastq.py command. Sequences were clustered via Uclust using the pick_otus.py command at 97% sequence similarity against the Greengenes Ribosomal database (http://greengenes.secondgenome.com/downloads). The reference file was customized to include the V4 region sequence from *Wigglesworthia* and *Sodalis* (these are absent from the Greengenes database). In order to remove possible chimeras and other PCR errors, all OTUs that did not align to the Greengenes database were excluded from the analysis. To remove OTUs with low read number, OTUs tables were filtered at 0.005% of the total number of reads [53]. The *G. pallidipes* samples were separately analyzed to determine the relationship between gut microbiota composition and tsetse trypanosome infection status, geographical location within one fly species. The relative abundance of each OTU was measured using the summarize_taxa.py script (http://qiime.org/scripts/summarize_taxa.html).

For α-diversity (species richness) calculations, datasets were rarefied to a depth of 15,001 sequences per sample. Alpha-diversity was calculated using the “observed species” metric with ten iterations at each sequencing depth. The number of OTUs at each sampling depth was averaged to make the rarefaction curves. To compare the α-diversity between *G. pallidipes* populations in different locations, a two-sample t-test with 1000 Monte Carlo permutations was used. Beta diversity (β-diversity) was calculated for comparisons between geographically distinct populations of *G. pallidipes* captured in geographically distinct locales. Jackknifed principal coordinate analysis (PCoA) and the unweighted UniFrac distance metric was used to visualize the difference between microbial communities form each population using ten jackknife replicates. We used an analysis for similarities (ANOSIM) test for group comparison between uninfected and infected flies from the same location using either unweighted or weighted unifrac distance matrices (*n* = 99 permutations).

### Genomic DNA extraction and PCR assays for symbiont quantification and trypanosome detection

Total DNA was extracted from ethanol preserved *G. pallidipes* midguts (*n* = 41) using the MasterPure Complete DNA Purification kit (Epicentre). Sterile water was used as a template during each batch of DNA extractions in an effort to detect bacterial contamination. Prepared DNA was quantified using a NanoDrop 2000 (Thermo Scientific).

Real time quantitative PCR (RT-qPCR) was performed using a CFX96 Real Time PCR Detection System (Bio-Rad). *Sodalis* specific *fliC* and *Wigglesworthia* specific *thiC* gene primers were used to quantify the relative abundance of these bacteria present within trypanosome infected and uninfected *G. pallidipes*. This was performed by comparing of experimental sample cycle threshold (C_t_) values to those derived from symbiont gene-specific internal standard curves [40]. *Wigglesworthia* cells are polyploid [40], thus in our analysis we measured genome copy number normalized to tsetse host *β-tubulin* gene copy number. The same method was applied to quantify *Sodalis.* RT-qPCR cycling conditions were as follows: initial denaturation at 95 °C for 8 min; 40 cycles of 95 °C for 15 s, 55 °C for 30 s, 95 °C for 1 min, and 55 °C for 1 min; and a melt curve of 55 °C to 95 °C in increments of 0.5 °C for 30 s. PCR primers are listed in Additional file 1: Table S2. All PCR assays were carried out in duplicate, and replicates were averaged for each sample. Negative controls were included in all amplification reactions.

## Additional file

Additional file 1: **Table S1.** Bacteria isolated from tsetse fly midguts using culture dependent techniques. **Table S2.** PCR primers used in this study. **Figure S1.** Average taxonomic composition and % abundance of environmentally acquired (top graph) and symbiotic bacteria (bottom graph) found in midguts of uninfected and trypanosome infected *G. pallidipes* collected in Nguruman, Kenya, Shimba Hills, Kenya and Murchison Falls, Uganda. (DOCX 508 kb)

## Abbreviations

AAT: Animal African trypanosomiasis
ANOSIM: Analysis for similarities
BHIB: Brain heart infusion with blood
C_t_: Threshold cycle
*G.*: *Glossina*
HAT: Human African trypanosomiasis
LB: Luria-Bertani media
MM: Mitsuhashi-Mahamarosh media
OTU: Operational taxonomic unit
PBS: Phosphate buffered saline
PCoA: Principal coordinate analysis
PGRP-LB: Peptidoglycan recognition protein LB
PM: Peritrophic matrix
rDNA: Ribosomal deoxyribonucleic acid
rRNA: Ribosomal ribonucleic acid
RT-qPCR: Reverse transcription quantitative PCR
