# Supplementary material for: Analysis of the gut-specific microbiome from field-captured tsetse flies, and its potential relevance to host trypanosome vector competence
Source: BMC Microbiol. 2018 Nov 23;18(Suppl 1):146. doi: 10.1186/s12866-018-1284-7 (PMC6251097; doi:10.1186/s12866-018-1284-7)
Supplement: Supplementary file 1 — Table S1. Bacteria isolated from tsetse fly midguts using culture dependent techniques. Table S2. PCR primers used in this study. Figure S1. Average taxonomic composition and % abundance of environmentally acquired (top graph) and symbiotic bacteria (bottom graph) found in midguts of uninfected and trypanosome infected G. pallidipes collected in Nguruman, Kenya, Shimba Hills, Kenya and Murchison Falls, Uganda. (DOCX 508 kb) [file 12866_2018_1284_MOESM1_ESM.docx]

**Table S1.** Bacteria isolated from tsetse fly midguts using culture dependent techniques.

| geographic origin | *Glossina* spp. | phylogenetic affiliation | closest relative according to BLASTn | # of isolates | E-value |
| --- | --- | --- | --- | --- | --- |
| Shimba Hills, Kenya | *G. pallidipes* | *Staphylococcus* spp. | *Staphylococcus saprophyticus* strain CD250Y | 2 | 0 |
|  |  | *Staphylococcus* spp. | *Staphylococcus* spp. MOLA 313 | 1 | 0 |
|  |  | *Staphylococcus* spp. | *Staphylococcus haemolyticus* strain B-16 | 1 | 0 |
|  |  | *Staphylococcus* spp. | *Staphylococcus sciuri* strain CD97 | 1 | 0 |
|  |  | *Staphylococcus* spp. | *Staphylococcus saprophyticus* strain CD250Y | 2 | 0 |
|  |  | *Staphylococcus* spp. | *Staphylococcus agnetis* strain 723 | 1 | 0 |
|  |  | *Xylella* spp. | Uncultured *Xylella* spp. clone I10-307 | 2 | 0 |
|  |  | *Agrococcus* spp. | *Agrococcus* spp. PLB053 | 1 | 0 |
|  |  | *Arthrobacter* spp. | *Arthrobacter* spp. OS-10C | 1 | 0 |
|  |  | *Enterobacter* spp. | *Enterobacter cloacae* strain Y219 | 2 | 1 |
|  |  | *Exiguobacterium* spp. | *Exiguobacterium acetylicum* strain N5 | 1 | 0 |
|  |  | *Exiguobacterium* spp. | *Exiguobacterium indicum* strain B3 | 1 | 0 |
|  |  | *Exiguobacterium* spp. | *Exiguobacterium* spp. P11 | 1 | 0 |
|  |  | *Bacillus* spp. | *Bacillus aryabhattai* strain fwz21 | 2 | 0 |
|  |  | *Bacillus* spp. | *Bacillus* spp. NQ18 | 1 | 0 |
|  |  | *Bacillus* spp. | *Bacillus flexus* strain NM25 | 1 | 0 |
|  |  | *Bacillus* spp. | *Bacillus* spp. Q-5 | 1 | 0 |
|  |  | *Bacillus* spp. | *Bacillus thuringiensis* serovar kurstaki str. HD73 | 1 | 2.5x10^-143^ |
|  | *G. brevipalpis* | *Bacillus* spp. | *Bacillus subtilis* strain CICC10200 | 1 | 0 |
|  |  | *Bacillus* spp. | *Bacillus megaterium* strain IARI-BC-13 | 1 | 0 |
|  |  | *Bacillus* spp. | *Bacillus aryabhattai* strain fwzb8 | 2 | 0 |
| Trans Mara, Kenya | *G. fuscipleuris* | *Staphylococcus* spp. | *Staphylococcus hominis* subsp. novobiosepticus strain ALK519 | 1 | 0 |
|  |  | *Bacillus* spp. | *Bacillus* spp. FJAT-17442 | 1 | 0 |
|  |  | *Kocuria* spp. | *Kocuria palustris* strain Abk-6 | 1 | 0 |
|  |  | *Microbacterium* spp. | *Microbacterium* spp. U1370-101126-SW187 | 1 | 0 |
|  |  | *Exiguobacterium* spp. | *Exiguobacterium* spp. AFB-18 | 1 | 0 |
|  |  | *Sinomonas* spp. | *Sinomonas atrocyanea* strain JN170 | 1 | 0 |
| Lake Victoria, Western Kenya | *G. fuscipes* | *Bacillus* spp. | *Bacillus horneckiae* type strain DSM 23495T | 1 | 0 |
|  |  | *Bacillus* spp. | *Bacillus horneckiae* strain RCTy1 | 1 | 4.3x10^-74^ |
|  |  | *Oceanimonas* spp. | *Oceanimonas* spp. GK1 | 1 | 0.068 |
|  |  | *Microbacterium* spp. | *Microbacterium foliorum* strain N1-12 | 1 | 0 |
|  |  | *Staphylococcus* spp. | *Staphylococcus haemolyticus* strain B-16 | 1 | 0 |
|  |  | *Arthrobacter* spp. | *Arthrobacter* spp. R-10(2010) | 1 | 9.7x10^-140^ |
|  |  | *Aeromonas* spp. | *Aeromonas* spp. DH-6 | 1 | 0 |
|  |  | *Providencia* spp. | *Providencia rettgeri* strain ALK058 | 1 | 0 |
|  |  | *Pantoea* spp. | *Pantoea dispersa* strain 1413 | 1 | 0 |

**Table S2. PCR primers used in this study.**

| gene | primer set | Tm (°C) |
| --- | --- | --- |
| *eubacterial 16s rRNA* (PCR) | F - 5'-AGAGTTTGATCCTGGCTCAG-3' | 54 |
|  | R - 5'-GGTTACCTTGTTACGACTT-3' | 55 |
| *tsetse β-tubulin* (RT-qPCR) | F - 5'-ACGTATTCATTTCCCTTTGG-3' | 54 |
|  | R - 5'-AATGGCTGTGGTGTTGGACAAC-3' | 55 |
| *Sodalis fliC* (RT-qPCR) | F - 5'-GAAGGTGTGATTTCTACGGGCAAAG-3' | 54 |
|  | R - 5'-GCGACTGGCTCAGGTTGGC-3' | 55 |
| *Wigglesworthia thiC* (RT-qPCR) | F - 5'-AAGTTATGATAGAAGGACCAGGAC-3' | 54 |
|  | R - 5'-CCCGGAGCAATATCAGTAGTTAG-3' | 54 |

**Figure S1**

**
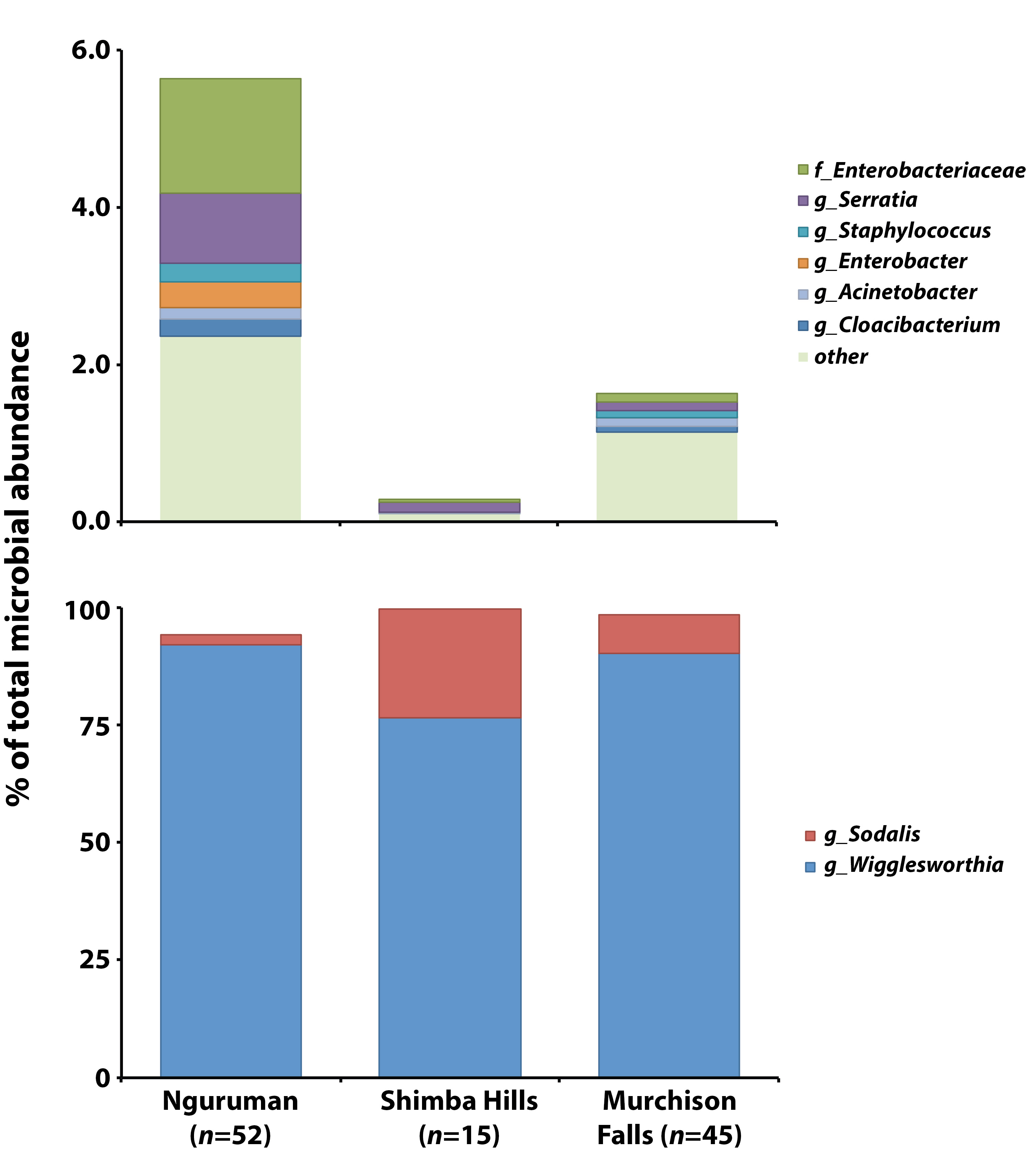
**
